# Long-Term Risk of Hepatic and Extrahepatic-Related Events After Direct Antiviral Therapy for Chronic Hepatitis C: A Prospective Long-Term Study Cohort

**DOI:** 10.3390/cancers17091528

**Published:** 2025-04-30

**Authors:** Luisa Cavalletto, Eleonora Bertoli, Claudia Mescoli, Camillo Aliberti, Maria Giovanna Quaranta, Loreta Kondili, Liliana Chemello

**Affiliations:** 1 UOC Clinica Medica 5, Regional Center for Liver Disease Outpatient Unit, Department of Medicine—DIMED, University of Padova, 35128 Padova, Italy; 2 Unit of Emergency Medicine, Department of Systems Medicine—DIDAS, University of Padova, 35128 Padova, Italy; eleonora.bertoli@aopd.veneto.it; 3 Unit of Pathology, Department of Medicine—DIMED, University of Padova, 35128 Padova, Italy; claudia.mescoli@aopd.veneto.it; 4 Unit of Radiology, Pederzoli Hospital Peschiera del Garda, 37019 Verona, Italy; camy.ali@libero.it; 5 Istituto Superiore di Sanità, Global Health Center, 00161 Rome, Italy; mariagiovanna.quaranta@iss.it (M.G.Q.); loreta.kondili@iss.it (L.K.)

**Keywords:** chronic hepatitis C, hepatitis C virus infection, hepatocellular carcinoma, direct-acting antivirals, hepatic and extrahepatic events

## Abstract

Novel direct antiviral-acting (DAA) molecules significantly improved efficacy and ameliorated outcomes of patients with chronic hepatitis C (CHC). The extensive use of DAA from 2015, due to large access to therapy, maximized rates of viral eradication with a safety profile in the majority of cases. Aims: We evaluated risk factors and the incidence of related clinical events and hepatocellular carcinoma (HCC) in cases with sustained virologic response (SVR) after DAA. We also aimed to apply a score assessment to identify the individual patient with unfavorable outcomes during an average follow-up (FU) of five years. Methods: In total, 470 cases consecutively recruited with CHC have been compared by non-invasive tests (NIT), as APRI, FORNS, FIB-4, LSPS, and transient elastography (TE) liver stiffness measurement (LSM), to identify cutoff related to major event onset. Results: Grouping of cases without or with related events development of both types hepatic (HE) (i.e., HCC or further cirrhosis decompensation or/with hospitalized septic state) or extrahepatic (EHE) (i.e., other tumors, bleeding, or thrombotic episodes and other organs pathologic conditions not liver related)allowed us to select the parameters to propose a novel risk stratification system (RISS) for the identification of the remnant individual patient’s risk for HCC occurrence, orthotopic liver transplant (OLT) need, or death during long-term follow-up (FU). Conclusions: Patients with cirrhosis and portal hypertension (PH) maintained a higher LSM mean value (>25 kPa), showed the lowest reduction of NIT scores, and developed events in 80/108 (74%) cases (67 and 13 of HE and EHE type), even after long-term successful DAA therapy. Furthermore, cases with RISS score ≥ 8 demonstrated a significant incidence of HCC (37/46, 80.4%) and a reduction in survival rate to 65.4% at 5-year FU.

## Simple Summary

After availability of oral direct-acting antivirals for treatment of chronic hepatitis C, it is required to define the long-term effectiveness of the extensive use of these novel molecules and, in particular, to analyze the outcome in term of morbidity and mortality, especially in senior cases or with advanced liver disease. The identification of the frail subject, which is not meant to have a significant change in life expectancy, even after HCV eradication, can give the opportunity to produce new algorithms predictive of unfavorable outcomes and can improve management of who maintains a high risk of hepatic- and extrahepatic-related events and, in particular, of hepatocellular carcinoma, liver transplantation, or death.

## 1. Introduction

The benefit of the antiviral therapy for chronic hepatitis C (CHC) with direct-acting antivirals (DAA) is not arguable, as these new molecules showed a wider spectrum of indication (i.e., also in patients with comorbidities or advanced liver disease and older age), sharing a good safety profile and a shorter oral administration (8–12 weeks) [1]. In addition to excellent tolerability, the DAA therapy demonstrated an overtime efficacy, obtaining HCV eradication in up to 90–95% of cases [2], regardless of previous resistance to interferon (IFN) and ribavirin (RBV) or HCV genotype, as pan-genotypic molecules [3].

These assumptions expected for a significant reduction not only of fibrosis (i.e., downstage of liver disease), but especially of rates of all complications related to cirrhosis, such as decompensation with ascites or encephalopathy, bleeding episodes of gastro-esophageal (GE) tract, or development of hepatocellular carcinoma (HCC) [4]. However, in some literature reports, a discrepancy has been highlighted with respect to these significant and expected results with DAA, especially for the most fragile patient categories. Furthermore, many papers focused on unexpected HCC onset [5,6,7] or higher rates of HBV reactivation [8] and, on description of severe drug interaction injury or increased rate of unfavorable outcome during the course or short term after, the DAA therapy [9].

Conversely, in many papers was underlined the beneficial role of DAA, particularly after attainment of sustained virologic response (SVR) [10,11], but in others was shown a remaining and sneaky risk of HCC onset or relapse after effective cure, especially in cases with advanced liver disease, with significant fibrosis or compensated cirrhosis stage [12,13]. Among different studies, the rate of HCC development after SVR ranges from 1.5 to 2.5/100 person-year (p-y) [14,15]. The factors involved in these differences were mostly attributable to the retrospective analysis and probably to the different characteristics of the study population, such as the estimated follow-up period (FU), the patient’s mean age, the liver disease stage, and the presence of hepatic nodules with undefined characteristics [16]. Beyond the aspect relating to liver disease, also the presence of comorbidities (i.e., obesity, diabetes, coinfections, cardiovascular disease, etc.) and risk habits (i.e., alcohol, drugs or tobacco consumption) has been linked to the higher risk of developing HCC [17,18]. Furthermore, interacting conditions during the long-term FU after DAA, which may be related to individual genetics, advanced liver disease without remission, or off-target effect of the used DAA, could likely have a decisive impact on the occurrence or recurrence of HCC. [19,20]. Some studies have investigated the improvement in scores or liver stiffness measurement (LSM) for liver fibrosis and portal hypertension (PH) staging after HCV eradication, documenting different risk categories for HCC incidence and some predictive factors associated with unfavorable outcomes [15,21]. However, limited data focusing on these potential predictors, when evaluated prospectively in the real-life practice of CHC cohorts successfully treated with DAA, are available.

The objective of our study was to define, at long-term FU, after DAA and achievement of SVR, the following goals: (a) the incidence rate of all types of related events, both hepatic (HE) or extrahepatic (EHE), occurred in the cohort; (b) the performance changes of liver fibrosis and PH non-invasive tests (NIT), as APRI, FORNS, FIB-4, LSPS, and of LSM by transient elastography (TE), obtained at basal time (before starting DAA therapy) and at least 36 months or at last FU after end of therapy (EOT); and (c) the identification of at-risk subjects for major clinical events (HCC occurrence, OLT, or death) incidence by a novel risk stratification system (RISS).

## 2. Methods

### 2.1. Study Design and Characteristics of Study Population

This prospective multicenter observational study was carried out in HUB outpatient liver centers at Padua University Hospital. The study protocol was approved by the local ethics committee, as an independent regional project, complies with the Helsinki rules and encloses written consent for all participants. Analyses included data of cases that complete a DAA therapy schedule from 2015 to 2022 and was restricted to participants who fulfilled requirements in terms of eligibility, interventions, and outcome assessment (per-protocol analysis).

The DAA schedule was chosen according to the EASL update guideline [22]. Antiviral treatment was administered to subjects aged between 18 and 75 years, in Italy, with priority for cases with histological features and/or LSM consistent with advanced chronic hepatitis or compensated cirrhosis Child–Pugh–Turcotte (CPT) class A5–6 and B7, after determination of serum viral loads by RT-PCR (Abbott Real-Time HCV, Chicago, IL, USA) and HCV genotyping (INNO-LIPA™ HCV-II, Innogenetics, Gent, Belgium).

All cases with absolute contraindication to DAA therapy, in particular, cirrhosis with recurrent decompensation, gastro-esophageal (GE) re-bleeding varices or active HCC, and patients with a poor motivation to comply and adhere to therapy or with a life expectancy of less than 5 years, were excluded.

In total, 520 cases were recruited, but 50 were excluded for previous solid organ transplantation (23 cases), for incomplete therapy schedules or FU period after EOT (23 cases), and for HCC or solid tumor onset before EOT (4 cases). All cases were fully investigated at basal time (within 1–3 months before DAA therapy start) and during a FU after EOT of at least 36 months, with laboratory and imaging examination to confirm the inclusion criteria to treatment. SVR was defined when HCV-RNA persisted undetectable at 12-week determination from EOT.

### 2.2. NIT for Estimation of Liver Fibrosis and Portal Hypertension

All cases underwent LSM (with a 50 Hz probe, ranging 1.5–75 kPa) by VCTE (Fibroscan^®^, Echosens, Paris, France). In particular, we also explored the following parameter to perform the formulas of liver NIT scores [23]: APRI = [(AST/upper limit NV AST) ×100]/number of platelets (10^9^/L), with cirrhosis stage defined at score ≥ 1.5 [24,25]; FORNS = 7.811 − 3.131 × ln [PLT count (10^9^/L)] × 0.781 ln [GGT (U/L)] + 3.467 × ln [age (years)] − 0.014 [cholesterol (mg/dL)], at score ≥ 6.9 [26]; FIB-4 = [age (years)] × AST (U/L)]/[number of platelets (10^9^/L)× RADQ ALT (U/L)], at score > 3.25 [27]; LSPS = LSM x spleen ø (cm)/PLT count (10^9^/L), with clinical significant PH at score > 3.5 [28].

### 2.3. Assessment of Clinical Liver Disease Stage

**Figure 1:**
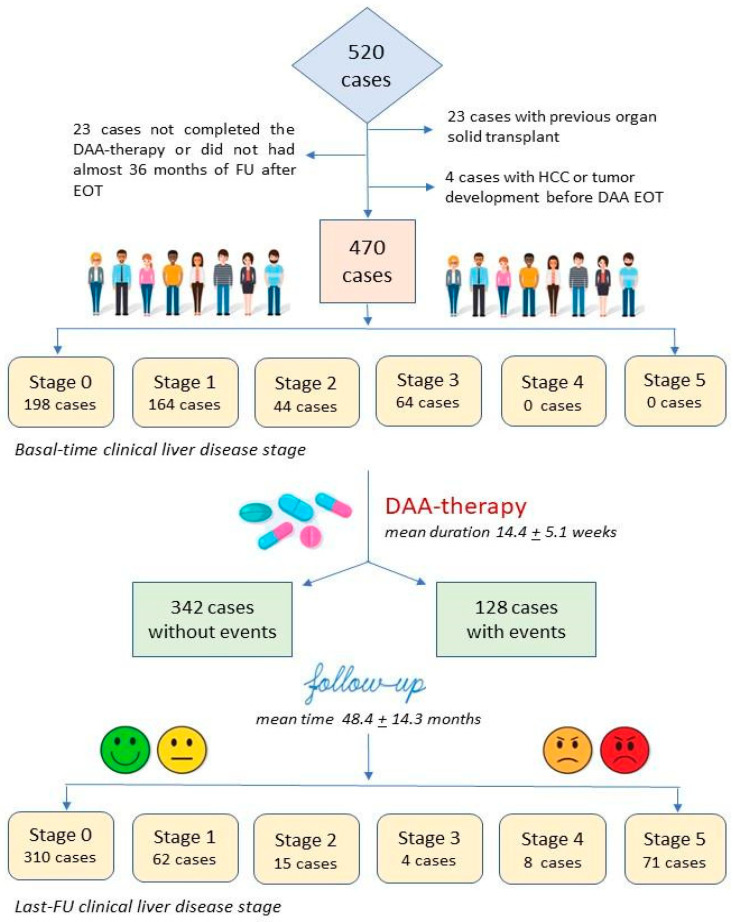
Flowchart of patients recruited, grouping by CLDS at basal time and at last FU and by cases without or with incidence of events during a 5-year FU.

The clinical liver disease stage (CLDS), applied in this analysis of cases with CHC receiving DAA therapy, aims to focus on some liver disease clinical stages, which remains not easy to differentiate, especially regarding the presence of cirrhosis [29], and as no measures or values by NIT scores or TE have a real defined range for the classification of cases with significant fibrosis or cirrhosis. It takes into account a six-stage level clinical approach, which refers to one of the following: stage 0 = mild fibrosis (cases without features on abdominal ultrasound or objective clinical examination significant for progressive liver disease and with LSM compatible with fibrosis stage F1–F2 according to METAVIR); stage 1 = significant fibrosis or early cirrhosis without sign of significant PH (i.e., absence of spleen enlargement or GE varices or varicose collateral circles); stage 2 = cirrhosis with presence of GE varices non-at-risk, at gastroscopy; stage 3 = cirrhosis with only one episode of resolved hepatic decompensation (i.e., ascites or GE bleeding); stage 4 = cirrhosis with recurrent episodes of decompensation; stage 5 = development of HCC or end-stage liver disease with further episodes of decompensation related to cirrhosis requiring OLT or resulting in death. This classification also allowed us to follow the change in clinical stage obtained, between clinical examination at basal time and that of the last FU in the cohort (see also Figure 1).

### 2.4. Risk Stratification Assessment of Events

The risk stratification system (RISS) is a set of conditions and clinical measures counted by a score and based on the probability of presence/absence of an event or clinical value, collected for each individual patient, which allows the identification of cases, which are mainly involved in the development of major events (onset of HCC, OLT, or death), obtained by statistical analysis of the study population and/or validation in different literature reports. The RISS scored *1 point* if presenting active smoking, alcohol consumption, comorbidities (consisting of cardiovascular disease or arterial hypertension, obesity or diabetes and MASLD, autoimmunity conditions, or previous or family history of cancers), male sex, age < 50 years, LSM values ≥ 15 kPa; *2 points* for aged > 70 years, LSM ≥ 25 kPa or with stage 2 or 3 at CLDS; and *3 points* for those with age between 50 and 70 years. This evaluation has a range score of 0–12 for each patient and consists of three categories: at low, moderate, or high risk in cases with score 0–4, 5–7, and ≥8 points, respectively, allowing the identification of susceptible or frail cases due to advanced liver disease and associated conditions, which increase the risk of all events occurring after DAA therapy, especially for HCC, OLT and death incidences.

### 2.5. Statistical Analysis

Mean value and standard deviation (SD) were applied to continuous variables showing a normal distribution, and the means were compared using the Student’s t-test for independent samples. The differences between categorical variables were evaluated with Pearson’s chi-square test or Fisher’s test, when appropriate. Differences between data of NIT scores and LSM obtained at different t-time were analyzed using ANOVA analyses of variance. Only significant parameters (*p* ≤ 0.05) obtained at univariate and multivariate Cox regression analyses were taken into consideration to identify predictors of related events. Analysis with Kaplan–Meier curves were used for estimation of events cumulative incidence or survival probability in cases with major events (HCC, OLT, or deceased) during a 5-year FU of the cohort. Comparison of the curves between/among groups was performed using Log-Rank test. These analyses were performed using the software STATA 9.0 (StataCorp LLC, Lakeway Drive, TX, USA) and MEDCALC software Ltd. (Ostend, Belgium).

## 3. Results

### 3.1. Clinical Characteristics and Changes During Long-Term FU

Figure 1 shows the flowchart of the study. Of 520 cases recruited, only 470 patients fully fit the inclusion criteria by a per-protocol analysis. Mean age was 59.4 ± 12.7 years, and 241 (51.3%) were male. Genotype HCV-1 was the most frequent infection type, with 288 (61.3%) cases. Patients received in 268 (57.0%) cases a DAA schedule with sofosbuvir (SOF), in 102 (21.7%) with glecaprevir plus pibrentasvir, in 64 (13.6%) with elbasvir plus grazoprevir, and in 36 (7.7%) with ritonavir-boosted paritaprevir plus ombitasvir and dasabuvir. Treatment mean duration was 14.4 ± 5.1 weeks. Basal time CLDS included 198, 164, 44, and 64 cases at stage 0, 1, 2, and 3, respectively, with 108 (23.0%) cases defined with PH (staged 2 or 3). No cases were included in stages 4 and 5, as these patients had absolute contraindication to be treated with DAA.

During a mean FU of 48.4 ± 14.3 months, we observed 310, 62, 15, 4, 8, and 71 cases with stage 0, 1, 2, 3, 4, and 5, respectively, with 79 (16.8%) cases shifted to stages 4 and 5. Furthermore, 52 (11.1%) cases were deceased, 13 (2.8%) received a liver transplant (OLT), and 40 (8.5%) were dropouts. In total, 128 (27.2%) patients developed events: 76 (59.4%) defined as HE (46 with HCC and 30 with advanced cirrhosis and PH stage, further decompensation episodes and development of abnormal coagulative, or septic state) and 52 of EHE type (22 with other tumor, 30 with bleeding or thrombosis not liver-related and pathological conditions regarding cardiology, neurology, or rheumatology)(see also Table S1, Supplementary Material).

**Figure 2:**
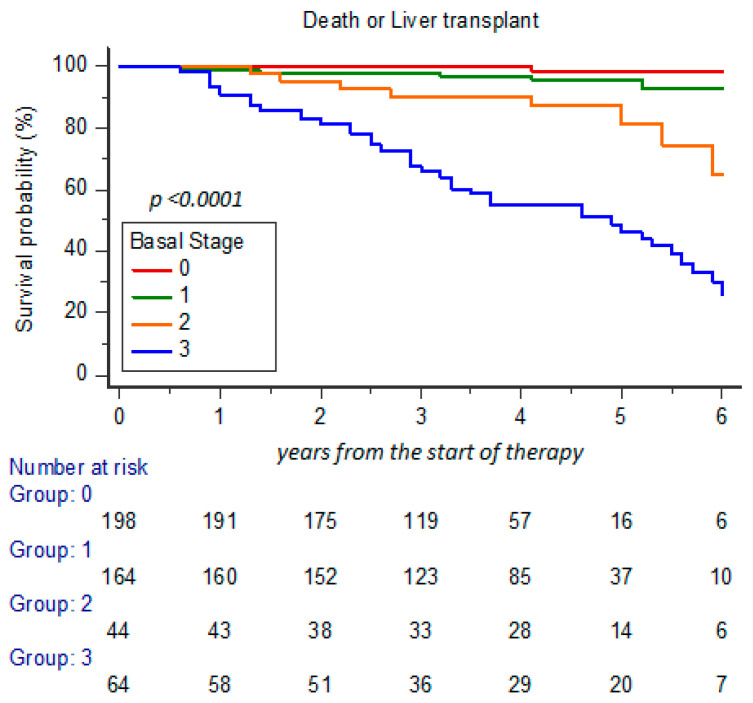
Survival probability according to basal time CLDS at 5-year FU estimation.

**Figure 3:**
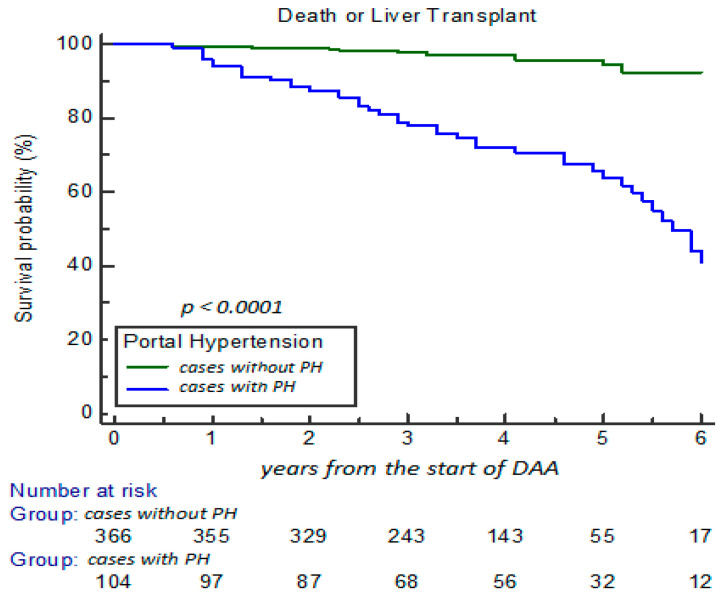
Survival probability according to cases without or with PH at 5-year FU estimation.

The survival probability analyzed (% cases with OLT or death) at 5-year FU showed a statistical difference among cases in relation to CLDS basal stage 0, 1, 2, and 3, with survival decreased to 98.9%, 95.1%, 79.5%, and 32.8%, respectively (*p* < 0.0001) (Figure 2). Moreover, the survival probability estimation was significantly reduced from 96.2% to 53.8%, particularly in relation to presence of PH at basal time (48/104 cases; 46.2%; with stage 2 and 3) in comparison to cases with stage 0 and 1 (14/366; 3.8%; *p* < 0.0001) (Figure 3).

### 3.2. Development of HE and EHE and the Impact on Clinical NIT Scores

The overall cohort was grouped in cases without incidence of events (342 cases; group 0) and with HE (76 cases; group 1) or EHE (52 cases; group 2) during the long-term FU (Table 1). Patients with HE had the majority of cases with smoking habit (60.5%), alcohol consumption (65,8%), and comorbidities (80.3%), and 69.7% were males. No differences appeared among groups in relation to HCV genotype infection; conversely, cases with events were mostly treated with SOF-based DAA schedules compared to cases without events (105/128 cases, 82.0% vs. 163/342, 47.7%, respectively, *p* < 0.001).

Considering the CLDS of the cohort at basal time in Table 1, 180/342 (52.6%) cases without events fell into the stage of mild fibrosis (0), and cumulatively, stages 0 and 1 accounted for 91.8% of these (*p* < 0.001). On the contrary, 67/76 (88.2%) cases with HE, with respect to 28 (8.2%) without events (group 0), were affected by cirrhosis with PH (*p* < 0.001). Moreover, the number of cases with CLDS stages 0 and 1 with respect to those with 2 and 3 appeared differently distributed with an inverse relationship, between cases with HE (group 1) and EHE (group 2) (all, *p* < 0.005). Finally, 46 cases developed HCC, and 53 (70%) cases were deceased in group 1, with respect to 9 cases (17.3%) in group 2 (*p* < 0.001).

Biochemical parameters, particularly those of liver function, confirmed significant differences among groups, especially of group 1 versus group 0 or 2. It should also be noted that between groups 0 and 2, there was a significant difference regarding a more advanced age, the presence of comorbidities, and the greater presence of cases with cirrhosis and PH and toward a decreased trend in the mean of platelet count and albumin level (*p* = 0.05) in the latter.

Table 2 presents the changes reported according to groups without and with events incidence during long-term FU by the NIT scores for staging of liver fibrosis and PH. Interestingly, all scores have shown a statistical difference between the values obtained at baseline compared to those of the last FU, although higher and significant values for rule-in the stage of cirrhosis are described in NIT scores of the group with HE (group 1). In the latter, the LSM obtained at the last FU remained at very high mean value (28.3 ± 14.4 kPa) compared to those of groups 0 (7.5 ± 4.1 kPa) and 2 (10.0 ± 7.4 kPa), as well as at FORNS and FIB-4 and LSPS scores: These data confirm the residual number of cases with advanced cirrhosis with PH and decompensated stage without signs of remission (staged 4 and 5) after successful DAA therapy.

During long-term FU, 46 cases had HCC occurrence; of these, 9 (19.6%) received OLT, and 28 (60.9%) were deceased, 2 cases after receiving OLT. Cases with HCC had significantly higher LSM in comparison to cases without tumor occurrence (36.1 ± 12.2 vs. 15.6 ± 10.3 kPa; *p* < 0.001), and also HCC incidence resulted in 31.7% and 68.3%, in cases with LSM > 15 kPa and ≥25 kPa, respectively, at basal time (*p* < 0.001). During long-term FU, cases that maintained clinical signs of PH and LSM > 25 kPa, showed HR of 7.34 (95%CI 3.77–14.31), with respect to the group with LMS < 25 kPa (HR: 0.14; 0.06–0.26) (*p* < 0.0001).

### 3.3. Assessment of Risk for Major Events Incidence at Long-Term FU (RISS Score)

To identify cases that would not benefit from the efficacy of DAA therapy, even after achieving SVR, we evaluate the risk of incidence of major events (onset of HCC and OLT or death) in the individual patient. The RISS scored into three categories, or score ranges, that defined a patient profile: at low (0–4 points), moderate (5–7), or high (≥8) risk. It was based on variables related to unfavorable outcomes, identified by statistical significance in our cohort, that allowed the plotting of curves for survival analysis.

**Figure 4:**
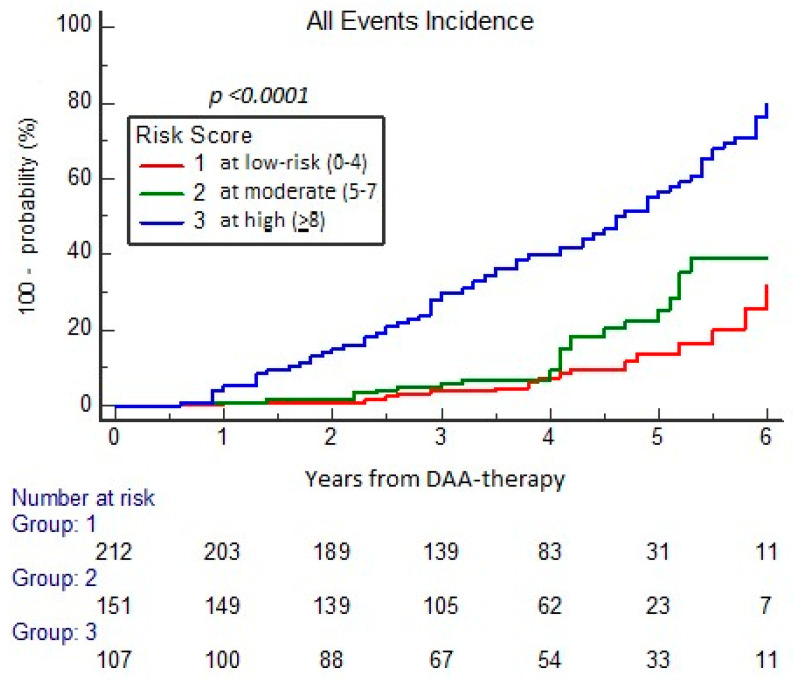
Probability of all events (HE and EHE type) according to categories of RISS.

Among 128 cases with events over 470 cases (27.23%) of the study cohort, those with high RISS score (≥8) had a significantly higher observed versus expected incidence, including 72.0% of all events, and showing, in comparison to moderate and low-risk groups, a HR of 4.2 (95% CI 2.80–6.40) versus 1.72 (1.27–2.64) and 0.57 (0.37–0.88), respectively (*p* < 0.001) (Figure 4).

**Figure 5:**
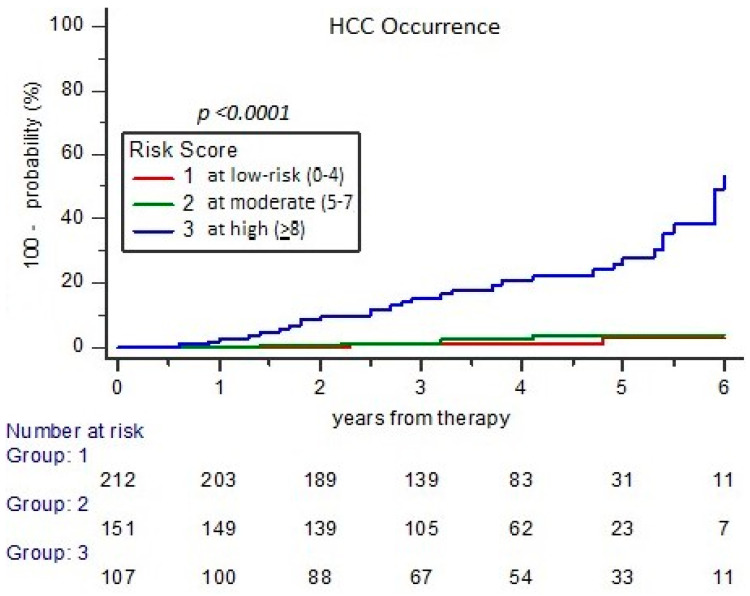
Probability of HCC occurrence according to risk categories of RISS.

Curves of cases with HCC occurrence (total cases 46/470; 9.8%) according to RISS profiles at high, moderate, or low risk had a distribution number of 34.6%, 3.3%, and 1.9%, respectively, showing a HR of 6.16 (95% CI 2.92–12.98), 1.78 (0.87–3.66), and 0.55 (0.27–1.14), respectively (*p* < 0.0001) (Figure 5). In the high RISS profile were included 37/46 (80.4%) cases with HCC onset during a 5-year FU.

**Figure 6:**
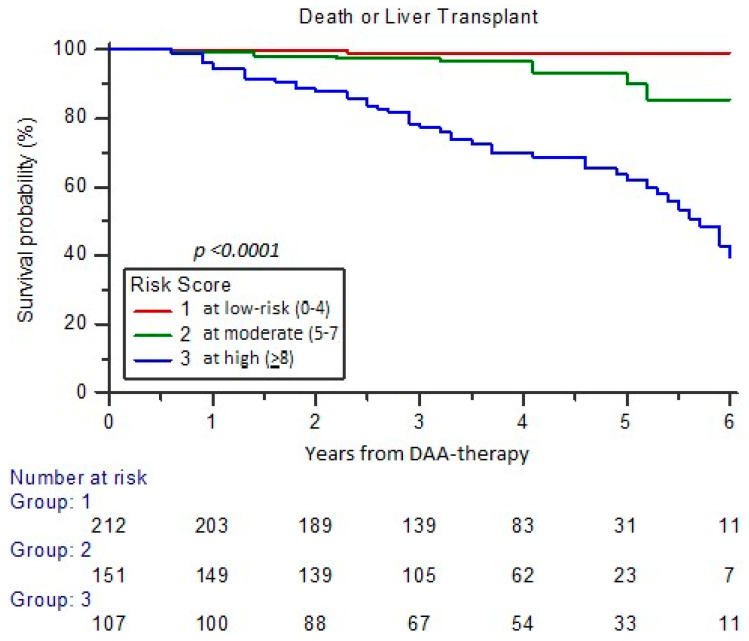
Probability of requiring OLT or dead according to risk categories of RISS.

The survival probability of the cohort analyzed, including cases requiring OLT or deceased (total number 62/470 cases; 13.2%) according to RISS score at low versus high risk, appeared to decrease from 98.6% to 55.1% at 5-year FU, with HR of 3.95 (95% CI 2.07–7.54) versus 0.19 (0.10–0.35), respectively (*p* < 0.0001) (Figure 6). The high RISS profile resulted in 48/62 (77.4%) of cases with OLT or dead at 5-year FU.

## 4. Discussion

This study aimed to present a series of events, occurring after a mean FU of 4.1 + 1.2 years, in a cohort of 470 patients with CHC achieving viral eradication, evaluated by per-protocol analysis. The extensive use of DAA to treat patients, even those vulnerable due to age, comorbidities, or more advanced liver disease, has led to the assumption that the effectiveness in avoiding liver-related complications could be achievable for all treated subjects. Unfortunately, over time, the use of DAA did not eliminate the incidence of major events, although in real-life practice, the high efficacy of HCV eradication translated into high effectiveness in reducing the HCC incidence risk [10]. Data from the PITER-HCV cohort in Italy [15] showed a HCC weighted cumulative incidence rate of 7.0% in cases with SVR, at 36-month FU, which was independently associated with male sex, increasing age, current alcohol use, HCV genotype 3, platelet count ≤ 120,000/μL, and albumin ≤ 3.5 g/dL.

Our study collected 128 (27.2%) cases with events, and the development of HCC had a 5-year cumulative incidence of 9.8%, but 272 (57.9%) cases staged with significant fibrosis or cirrhosis, including 108 cases (23%) with PH. Cases with HE included 46 cases with HCC and 30 with advanced cirrhosis, of which 88.2% had PH. It is evident that our cohort, being treated between 2015 and 2017, was affected by a priority treatment phase in favor of cases with advanced cirrhosis CPT-A6 and -B7 classes. The greater predisposition of these cases to unfavorable outcomes after DAA has been supported by many studies of literature [30]. In particular, of 350 cases with cirrhosis described in the Asian population, 70 patients (20%) developed HCC, and 9.1% decompensated during therapy, especially in the group with CPT-B or C, which represented 44.3% of the study population [31]. Instead, considering an Italian study with 138 cases aged ≥ 70 years, 70% of cases had compensated cirrhosis, and among these, 8 cases (14.5%) developed HCC [32].

To raise awareness of the careful and responsible use of DAA therapy, in particular in *frail-to-treat* patients, since exposition can lead to some related events, the objective of having useful tools for predicting its incidence is desirable. In our study, we classified both the clinical aspect of liver disease, proposing a staging classification or CLDS, similar to others in literature [29], which allowed us to identify the subjects most predisposed to major events, but it also contributed to a risk stratification system with three-profile (the RISS score) for the analysis of the morbidity and mortality of the cohort.

With regard to the comparison between cases with EH and EHE, the greater frequency of males in the group with HE was highlighted, but no influence of the HCV genotype infection among groups was seen, and a significant relationship between the use of SOF-based schedules was linked to occurrence of major events (see Table S1, Supplementary Materials). This phenomenon remains difficult to explain, but notice also by other contributions in the literature, where off-target effects related to the DAA use [19] or by drug-to-drug interactions [9] were hypothesized, in particular with regard to a greater incidence/recurrence of HCC [5,6]. Following and larger studies nullify this relation, which was attributed to a cohort bias due to higher number of cases mainly at risk of HCC onset for presence of cACLD [33,34]. In our cohort, we had a cumulative incidence of HCC of 1.6/100 p-y at 5-year FU estimation, which resulted similarly to many other studies of literature [14,15,16,17,18,19,20,21,22,23,24,25,26,27,28,29,30,31,32,33,34,35].

NIT scores varied significantly from basal, among the groups described, at last FU, and it is undoubtedly an expected finding due to HCV eradication. This goal, which drives the effectiveness of treatment, particularly in cases with significant fibrosis or early cirrhosis, is confirmed by a large retrospective cohort in 40,654 cases treated with DAA versus untreated cases for insured patients with CHC, which showed a significant improvement in outcomes, with a higher overall survival among cases with SVR [30]. In contrast, the greatest number of major events occurs in relation to cases with cirrhosis with PH in 88.2%, which also showed a higher mean of LSM (>25 kPa) and the lowest reduction of NIT scores, even after long-term success of DAA therapy.

The Baveno VII “*rules of 5*” for LSM successfully help to recognize cases with compensated cirrhosis and PH [36], and also in our RISS score, cases with LSM > 15 and ≥25 kPa identified subjects at risk of major events (HCC, OLT, or death), when combined to other parameters (i.e., risk habits, comorbidities, gender and age), that showed a differentiate weight as variables defined strong or weak for HCC correlation.. The novel RISS demonstrated a good prognostic accuracy, with HR 4.24 (95% CI 2.80–6.48) between cases with high versus low (0.57; 0.37–0.88) or moderate-risk (1.72; 1.12–2.64) categories for incidence of all type of events (*p* < 0.0001). The RISS score ≥ 8 showed a significant correlation with incidence of all events (107/128 cases; 83.6%) and HCC (36/46 cases; 80.4%) and identified the category of cases with a reduction in survival rate to 65.4% at 5-year FU.

These original data add knowledge supporting the relevance of clinical classification of liver disease, which is closely related to the prognosis and survival of cases with CHC successfully treated with DAA, and the proposed RISS could effectively help us with a tailored post DAA FU, for early diagnosis of HCC in individuals who remain at risk.

## 5. Conclusions

Nowadays, serious complications related to cirrhosis and portal hypertension inevitably maintain the related morbidity and mortality after DAA therapy, despite viral eradication. Our study aimed to focus on the development of adverse outcomes, which may occur after DAA exposure. In particular, hepatic-related events accounted for the largest number of cases staged with advanced liver disease, reaching 88.2%. Only through detailed staging of fibrosis by evaluation of NIT and LSM could we accurately predict the risk of major events onset. All cases with RISS score ≥ 8 had higher LSM (>25 kPa) and lower reduction of NIT scores, even after long-term successful DAA therapy, and had shown a risk of HCC in 80.4% of cases and a reduction in survival rate to 65.4% at 5 years.

## Figures and Tables

**Table 1:** Characteristics of study population according to development of HE or EHE.

	Cases Without Events n. 342 (72.7%)	*p*-Level 0 vs. 1	Cases with HE n. 76 (16.2%)	*p*-Level 1 vs. 2	Cases with EHE n. 52 (11.0%)	*p*-Level 0 vs. 2
**Characteristics**	**Group 0**		**Group 1**		**Group 2**	
N° males (%)	164 (47.9%)	<0.001	53 (69.7%)	0.01	24 (31.6%)	ns
Age (mean years ± SD)	57.6 ± 12.9	ns	59.6 ± 11.4	ns	63.6 ± 12.9	<0.005
BMI (Kg/m^2^)	25.1 ± 4.0	<0.05	26.8 ± 4.3	ns	25.4 ± 4.1	ns
Smoking habit	155 (45.3%)	<0,05	46 (60.5%)	0.05	22 (42.3%)	ns
Alcohol consumption	175 (51.2%)	<0.05	50 (65.8%)	ns	25 (48.1%)	ns
Comorbidities	196 (57.3%)	<0.001	61 (80.3%)	ns	39 (75.0%)	<0.05
HCV genotype 1 infection	212 (61.9%)	ns	44 (57.9%)	ns	32 (61.5%)	ns
DAA schedule SOF-based	163 (47.7%)	<0.001	68 (89.5%)	0.01	37 (71.1%)	<0.001
**Basal Time Clinical Liver Disease Stage**						
Mild fibrosis (stage 0)	180 (52.6%)	<0.001	1 (1.3%)	<0.001	17 (32.7%)	<0.001
Significant fibrosis or cirrhosis without EV (stage 1)	134 (39.2%)	<0.001	8 (10.5%)	<0.001	22 (42.3%)	ns
Cirrhosis with EV (stage 2)	24 (7.0%)	0.01	13 (17.1%)	<0.005	7 (13.4%)	ns
Cirrhosis with only one known decompensation (stage 3)	4 (1.2%)	<0.001	54 (71.1%)	<0.001	6 (11.5%)	0.001
**Development of Major Events during FU**						
Cases with HCC	0	*--*	46 (60.5%)	*--*	0	*--*
Cases with OLT or/and dead	0	*--*	53 * (70.0%)	<0.001	9 (17.3%)	*--*
**Biochemical Laboratory Parameters**						
Hemoglobin (g/L)	141.8 ± 17.7	<0.001	129.3 ± 20.9	0.01	137.6 ± 16.6	ns
White cell count (mm^3^)	6.1 ± 1.9	<0.001	4.5 ± 1.9	<0.005	5.9 ± 1.8	ns
PLTS count (mm^3^)	204.8 ± 72.6	<0.001	96.5 ± 68.4	<0.001	179.7 ± 87.1	0.05
INR ratio	1.08 ± 0.25	<0.001	1.22 ± 0.21	<0.005	1.11 ± 0.26	ns
Total bilirubin (μmol/L)	11.5 ± 5.7	<0.001	22.5 ± 9.9	0.001	11.1 ± 5.9	ns
ALT (U/L)	93.9 ± 89.5	<0.005	70.5 ± 48.1	ns	90.7 ± 67.4	ns
Gamma-GT (U/L)	57.2 ± 60.4	0.001	99.6 ± 86.9	ns	71.8 ± 63.7	ns
Total protein (g/L)	74.6 ± 5.0	ns	74.5 ± 6.7	ns	73.7 ± 7.9	ns
Albumin (g/L)	41.5 ± 4.2	<0.001	35.9 ± 4.8	0.005	39.3 ± 4.8	0.05
Gamma-globulin (g/L)	14.6 ± 4.6	<0.001	20.7 ± 6.1	<0.005	15.2 ± 4.8	ns
Alpha-fetoprotein (μg/L)	8.2 ± 17.6	<0.05	22.4 ± 50.6	ns	12.5 ± 19.9	ns

* Two cases were transplanted and afterwards deceased. “ns”, not significant.

**Table 2:** Staging of liver fibrosis and PH by NIT scores. Comparison of values obtained at basal time and at last FU according to development of HE or EHE in the cohort.

	Cases Without Events	*p*-Level0 vs. 1	Cases with HE	*p*-Level1 vs. 2	Cases with EHE	*p*-Level0 vs. 2
Liver NITs	Group 0		Group 1		Group 2	
N° cases (%) *	309 (90%)		60 (79%)		40 (77%)	
LSM ** at BT ***	11.7 ± 7.7	<0.001	32.6 ± 12.0	<0.01	15.2 ± 9.1	ns
LSM at last FU	7.5 ± 4.1	<0.001	28.3 ± 14.4	<0.005	10.1 ± 7.4	ns
APRI at BT	1.0 ± 0.9	<0.001	2.5 ± 1.8	<0.001	1.4 ± 1.2	<0.05
APRI at last FU	0.4 ± 0.2	<0.001	1.6 ± 1.2	<0.01	0.6 ± 0.5	ns
FORNS at BT	8.1 ± 1.5	<0.001	11.5 ± 1.6	<0.01	9.2 ± 1.8	<0.01
FORNS at last FU	7.3 ± 1.2	<0.001	10.9 ± 1.7	<0.001	8.2 ± 1.7	<0.01
FIB-4 at BT	2.5 ± 1.4	<0.001	8.5 ± 4.3	<0.001	4.4 ± 2.7	<0.005
FIB-4 at last FU	1.7 ± 0.7	<0.001	6.9 ± 4.4	<0.001	3.0 ± 2.1	<0.05
LSPS at BT	1.0 ± 0.8	<0.001	8.1 ± 5.0	<0.001	1.8 ± 1.6	<0.05
LSPS at last FU	0.6 ± 0.5	<0.001	6.9 ± 5.4	<0.001	1.0 ± 0.9	ns

* Only cases with complete data for all scores were analyzed. ** TE units (kPa). *** BT, basal time. “ns”, not significant.

## Data Availability

All the data used for the study are stored in a database with security measures compliant with privacy legislation and can be consulted in an anonymous form, upon request to the researchers who conducted the study.

## Supplementary Materials

The following supporting information can be downloaded at: https://www.mdpi.com/article/10.3390/cancers17091528/s1, Table S1: Type of LR or not-LR events occurred in the cohort during a 5-year FU.

## Abbreviations

ALT	alanine aminotransferase
APRI	aspartate aminotransferase to platelet ratio index
AST	aspartate aminotransferase
BMI	body mass index
cACLD	compensated advanced liver disease
CI	confidential interval
CHC	chronic hepatitis C
CLDS	clinical liver disease stage
RISS	Risk stratification system
DAA	direct-acting antiviral
DILI	drug-induced liver injury
EHE	extrahepatic-related events
EOT	end of therapy
EV	esophageal varices
FIB-4	fibrosis-4 index for detection of liver fibrosis or cirrhosis
FORNS	staging of liver fibrosis or cirrhosis according to Forns’ formula
GGT	gamma-glutamil transferase
GE	gastro-esophageal
HCC	hepatocellular carcinoma
HCV	human hepatitis C virus
HE	hepatic-related events
HR	hazard ratio
LSM	liver stiffness measures
LSPS	liver stiffness platelet score
MASLD	metabolic dysfunction-associated steatotic liver disease
NIT	non-invasive test
OLT	orthotopic liver transplantation
PH	portal hypertension
SD	standard deviation
SOF	sofosbuvir
SVR	sustained virologic response
TE	transient elastography
