# Supplementary material for: Long-Term Risk of Hepatic and Extrahepatic-Related Events After Direct Antiviral Therapy for Chronic Hepatitis C: A Prospective Long-Term Study Cohort
Source: Cancers (Basel). 2025 Apr 30;17(9):1528. doi: 10.3390/cancers17091528 (PMC12071134; doi:10.3390/cancers17091528)
Supplement: Supplementary file 1 [file cancers-17-01528-s001.zip › cancers-3506360-supplementary.pdf]

**Supplementary Material**

**Table S1**: Type of LR or not-LR events occurred in the cohort during a 5-year FU

| Type of event | Hepatic related events (HE) (76 cases) | Extrahepatic related events (EHE) (52 cases) |
|---|---|---|
| **HCC occurrence** | **46** | **0** |
| **Cirrhosis with PH** | **13** (9 with alcohol or substances abuse and 4 DILI) | **0** |
| **Others solid tumors** | **0** | **22** (5 with lymphomas, 4 thyroids, 3 pancreas, 3 kidneys, 3 esophageal, 3 breasts and 1 pulmonary) |
| **Coagulative abnormalities** | **8** (3 with portal thrombosis, 4 GE varices bleeding and 1 cerebral hemorrhage for very low INR) | **11** (5 with venous thrombotic events and 6 bleeding not related to PH or GE varices ) |
| **Cardiological events** | **0** | **4** (4 with heart arrhythmias and PM implantation) |
| **Sepsis/severe Infections** | **9** (3 with cholangitis, 4 systemic mycosis and 2 abscesses) | **9** (4 with pneumonia, 2 erysipelas and 3 urological) |
| **Other conditions with autoimmunity** | **0** | **6** (4 with rheumatological and 2 dermatological) |
